# 261. Adaptive Immunosuppression in Children with Septic Shock

**DOI:** 10.1093/ofid/ofad500.333

**Published:** 2023-11-27

**Authors:** Diego A Cruz Vidal, Jennifer Muszynski, Ian Goldthwaite, Leahmaria Ayad, Andrew Snyder, Jill Popelka, Josey Hensley, Lisa Steele, Katherine Bline, Mark Hall

**Affiliations:** Nationwide Children's Hospital, Columbus, Ohio; Nationwide Children's Hospital, Columbus, Ohio; Nationwide Children's Hospital, Columbus, Ohio; Nationwide Children's Hospital, Columbus, Ohio; Nationwide Children's Hospital, Columbus, Ohio; Nationwide Children's Hospital, Columbus, Ohio; Nationwide Children's Hospital, Columbus, Ohio; Nationwide Children's Hospital, Columbus, Ohio; Nationwide Children's Hospital, Columbus, Ohio; Nationwide Children's Hospital, Columbus, Ohio

## Abstract

**Background:**

Septic children can develop a severe compensatory anti-inflammatory response, termed “immunoparalysis (IP)”, which is strongly associated with adverse outcomes. IP can be defined as a reduced ability of innate immune cells to produce TNFα when whole blood is stimulated ex vivo with LPS (a TNFα response < 200 pg/ml). Adaptive (lymphocyte-based) immune function is poorly understood in IP. We hypothesize that septic children with IP will also have suppressed lymphocyte cytokine production capacity and reduced lymphocyte populations.

**Methods:**

Single-center, prospective, observational study of children (< 18 yrs) with septic shock. Subjects were enrolled and sampled < 48 hrs from shock onset. Ex vivo whole blood stimulation assays were used to evaluate innate (LPS-induced) and adaptive (PHA-induced) cytokine production capacities (Fig1). Cell populations were measured by flow cytometry (Fig2). Identical sampling and processing were done in a cohort of healthy control children. Data represent median (IQR).

**Figure 1.**
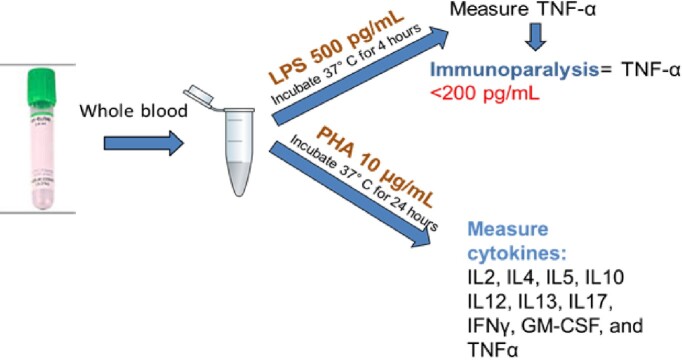
Ex-vivo stimulation assays

TNFα production after lipopolysacharide (LPS) stimulation (innate immune response) is measured by chemiluminescence (Siemens Healthcare Diagnostics). Cytokine production after phytohemagglutinin (PHA) stimulation (adaptive immune response) is measured by multiplex assay (Biorad).

Table 1

**Figure:**
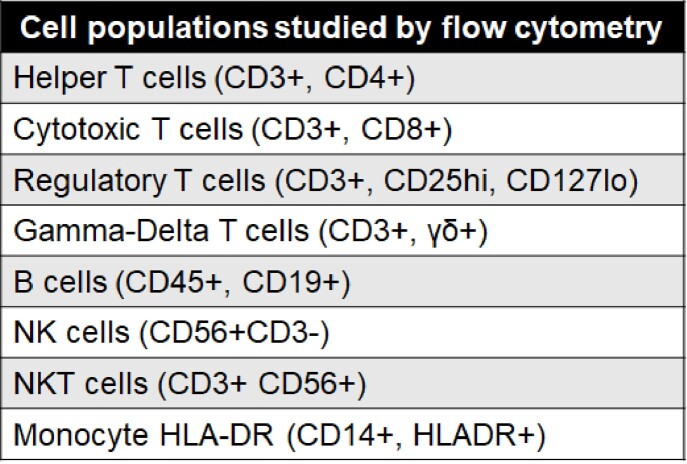

Whole blood was stained within 30 minutes of collection and included the cell populations shown above. Samples were acquired on the cytometer within a week of staining and analyzed using FlowJo 10.8.0 software.

**Results:**

30 children with septic shock (15 [8,16] yrs; 64% male, 3% mortality) and 11 healthy controls (6 [3,8] yrs; 54% male) were enrolled. Among septic subjects, length of stay was 5 [2,18] days in the ICU and 17 [6, 38] days in the hospital. Etiologies and sites of infection are shown in Fig3.

Septic subjects had lower ex vivo PHA-induced cytokine production (Fig4), and relative lymphopenia by flow cytometry vs healthy controls, especially double negative-T cells (3.7% vs 8.4%; p=0.03) and γδ-T cells (2.9% vs 7.6%; p=0.03).

12 septic subjects had IP. Rates of viral detection (50% vs 11%; p=0.03) and viral-bacterial co-detection (63% vs 9%; p=0.04) were higher in the IP group vs non-IP septic subjects. Subjects with IP had lower monocyte HLA-DR expression but no differences in lymphocyte populations. PHA-induced production of Th1 cytokines was lower in those with IP, while production of Th2 cytokines was preserved (Fig5).

**Figure:**
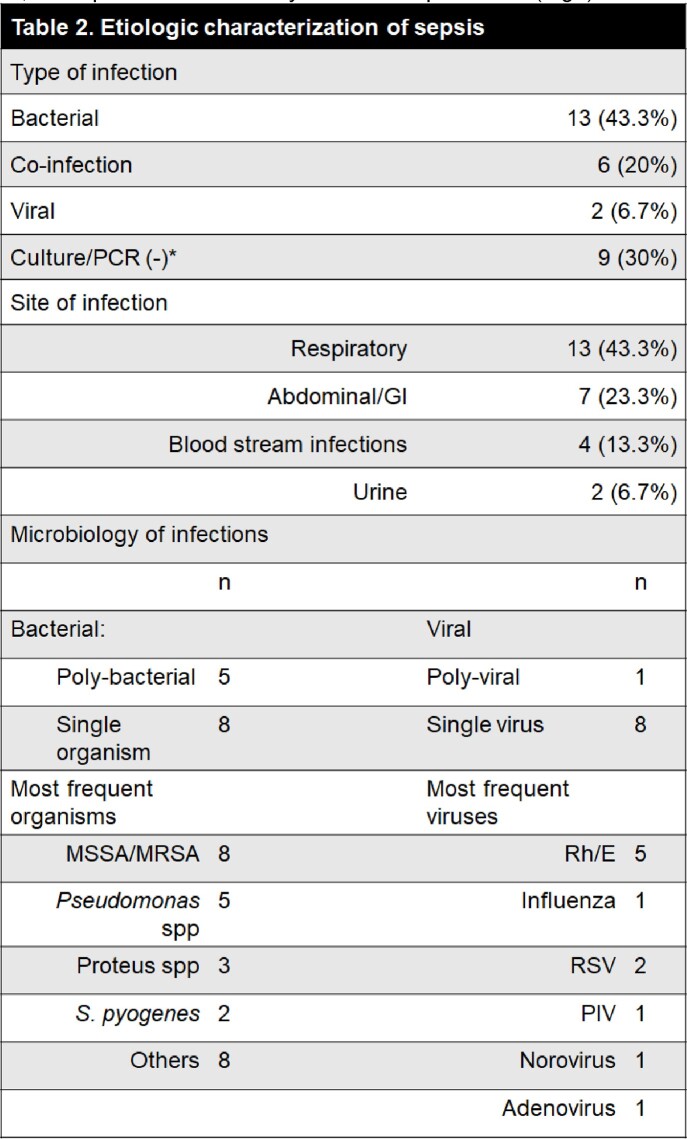

*Subjects where included in analysis if treated as presumed bacterial infections. Abbreviations: PCR: Polymerase chain reaction; GI: Gastrointestinal; MSSA: Methicillin susceptible S aureus; MRSA: Methicillin resistant S aureus; Rh/E: Rhinovirus/Enterovirus; RSV: Respiratory Syncytial virus; PIV: Parainfluenza Virus.

**Figure 4.**
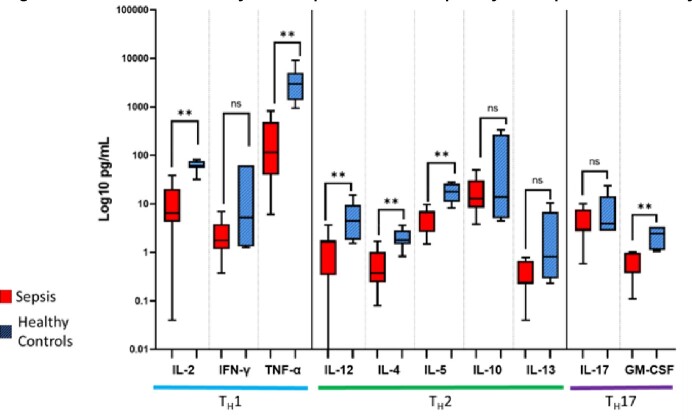
PHA-induced cytokine production capacity in sepsis vs healthy controls

Comparisons done by Mann-Whitney U test. Asterisks represent p-value <0.05.

**Figure 5.**
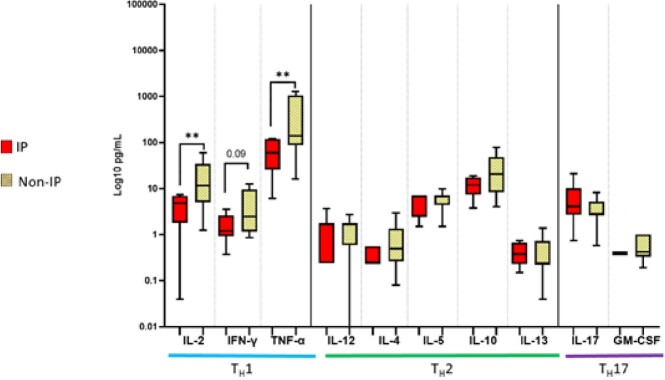
PHA-induced cytokine production capacity in children with and without immunoparalysis (IP)

Comparisons done by Mann-Whitney U test. Asterisks represent p-value <0.05.

**Conclusion:**

Children with septic shock have early innate *and* adaptive immune suppression. Immunoparalysis was associated with skewing to a Th2 profile of cytokine production, and with the presence of viral infections. This approach has the potential to drive enrollment into clinical trials of personalized immunomodulation in this population.

**Disclosures:**

**Mark Hall, MD FCCM**, Abbvie: Advisor/Consultant|Kiadis: Honoraria|Partner Therapeutics: complementary study drug Sobi for a clinical trial for which I am the Principal Investigator|Sobi: Complementary study drug for a clinical trial for which I am the Principal Investigator

